# 新型多羟基硅胶固定相的制备及其对褐藻胶寡糖的分离应用

**DOI:** 10.3724/SP.J.1123.2025.03006

**Published:** 2026-04-08

**Authors:** Ruiwen JIAO, Dandan ZHANG, Xingyuan HU, Wanting ZHU, Xiang LI, Yidi CAI, Dandan REN, Qiukuan WANG, Long WU, Hui ZHOU

**Affiliations:** 1.大连海洋大学食品科学与工程学院，辽宁 大连 116023; 1. College of Food Science & Engineering，Dalian Ocean University，Dalian 116023，China; 2.国家海藻加工技术研发分中心，辽宁 大连 116023; 2. National R & D Branch Center for Seaweed Processing，Dalian 116023，China; 3.辽宁水产品加工及综合利用重点实验室，辽宁 大连 116023; 3. Key Laboratory of Aquatic Product Processing and Utilization of Liaoning Province，Dalian 116023，China

**Keywords:** 超支化聚甘油, 亲水色谱固定相, 亲水机理, 褐藻胶寡糖, hyperbranched polyglycerol, hydrophilic chromatographic stationary phase, hydrophilic mechanism, alginate oligosaccharides

## Abstract

超支化聚甘油（hyperbranched polyglycerols，HPG）是一种具有高度支化结构的聚合物，分子呈三维球状，拥有大量的端羟基。采用HPG在二氧化硅表面聚合制备了一种多羟基硅胶亲水色谱固定相（HPG-Sil 3）。元素分析、红外光谱、热重分析、氮气吸附脱附等温线测定等表征结果表明HPG成功接枝在硅胶表面。以胸腺嘧啶、尿嘧啶、次黄嘌呤和腺苷为标准分析物，考察了乙腈含量、缓冲盐浓度和pH值等因素对溶质保留因子的影响，探讨了固定相的保留机理。结果表明HPG-Sil固定相符合亲水色谱机理，是一种亲水模式色谱柱。将HPG-Sil固定相应用于褐藻胶寡糖分离发现，HPG-Sil固定相具有良好的亲水分离效果和稳定性，在极性化合物色谱分离领域具有较高的应用潜力。

## Abstract (en)

Hyperbranched polyglycerols （HPG） represent an emerging class of dendritic polyethers characterized by a highly branched， three-dimensional architecture and a multitude of terminal hydroxyl groups. This distinctive structural configuration confers exceptional hydrophilicity， biocompatibility， and a high density of modifiable surface functionalities， thus establishing HPG as highly promising materials for the development of advanced chromatographic stationary phases. Their application is particularly relevant for the separation of polar and hydrophilic analytes， which has long posed a significant challenge in conventional reversed-phase liquid chromatography. The integration of such hyperbranched polymers with robust inorganic substrates， such as silica gel， has recently gained traction as a sophisticated materials strategy. This approach synergistically combines the superior mechanical strength and pressure resistance of the inorganic matrix with the rich surface chemistry and tunable hydrophilicity of the polymer， thereby addressing critical limitations of traditional stationary phases in hydrophilic interaction liquid chromatography （HILIC） applications.In the present study， a novel HILIC stationary phase， designated HPG-Sil 3， was synthesized through the in-situ ring-opening polymerization of glycidol monomers from the surface of aminopropyl-functionalized silica microparticles. The synthesis commenced with a meticulous silanization step to graft （3-aminopropyl）triethoxysilane （APTES） onto the silica surface， thereby introducing a uniform layer of primary amine groups. These amine functionalities served as initiation sites for the subsequent grafting-from polymerization， ensuring the formation of a covalently anchored， robust HPG layer. This covalent immobilization strategy is critical for mitigating stationary phase degradation and polymer leaching under prolonged chromatographic use， thereby guaranteeing long-term operational stability. The successful fabrication of the HPG-Sil 3 material and its physicochemical properties were thoroughly characterized using a suite of analytical techniques. Elemental analysis indicated a substantial increase in carbon and hydrogen content post-modification， providing quantitative evidence of organic polymer grafting. Fourier-transform infrared （FTIR） spectroscopy further corroborated this result， revealing signature absorption bands associated with the stretching vibrations of O-H and C-O-C ether linkages， which are characteristic of the HPG polyether structure. Thermogravimetric analysis （TGA） demonstrated the material’s excellent thermal resilience， with the onset of HPG decomposition occurring above 200 ℃， a temperature window far exceeding the operational range of typical HILIC analyses. Textural properties， evaluated via nitrogen physisorption， showed a predictable decrease in specific surface area and pore volume relative to the unmodified silica substrate. This reduction is attributed to the partial filling of the mesoporous silica network by the grafted HPG chains. Importantly， the material retained a sufficiently open porous structure to facilitate efficient mass transfer of analytes during chromatographic runs. To systematically investigate the chromatographic behavior and retention mechanism of the HPG-Sil 3 phase， a set of model polar compounds， including thymine， uracil， hypoxanthine， and adenosine， was selected. The influence of critical mobile phase parameters on the analyte retention factor （*k*） was examined， including the acetonitrile （ACN） content， the concentration of ammonium acetate buffer， and the buffer pH. The observed retention trends were unequivocally indicative of a dominant HILIC mechanism. A pronounced increase in retention with increasing ACN content was observed for all analytes， consistent with the enhanced partitioning of solutes into a water-rich layer immobilized on the hydrophilic stationary phase surface. Conversely， an increase in buffer concentration led to a decrease in retention， a phenomenon explained by the competitive adsorption of buffer ions with the analytes for polar interaction sites on the HPG layer. Furthermore， the retention of ionizable analytes， namely hypoxanthine and adenosine， was demonstrably influenced by the buffer pH， as pH variations alter their ionization state and thus their overall hydrophilicity and interaction strength with the stationary phase. Under the optimized chromatographic conditions， all four model analytes were baseline separated within a 10-min runtime， exhibiting excellent peak symmetry and high repeatability. The practical utility of the HPG-Sil 3 stationary phase was further demonstrated through the efficient separation of alginate oligosaccharide homologs with degrees of polymerization （DP） ranging from 2 to 7. The elution order followed increasing DP， which aligns perfectly with the HILIC retention principle， as larger oligosaccharides possess more hydroxyl groups and exhibit stronger hydrophilic interactions. The stationary phase also demonstrated remarkable operational stability， with no observable changes in retention times or chromatographic efficiency over 10 consecutive injections， underscoring its robustness for routine analytical applications. In conclusion， the HPG-Sil 3 stationary phase synthesized in this work exhibits outstanding hydrophilic separation performance， a well-understood HILIC retention mechanism， and excellent long-term stability. These attributes position it as a highly competitive and promising material for the analysis of a wide array of polar compounds， including nucleosides， carbohydrates， and polar pharmaceuticals， across diverse fields such as metabolomics， biopharmaceutical analysis， and food chemistry. Future research will focus on the precise control of polymer parameters， including molecular weight， branching density， and post-functionalization， to fine-tune the properties of the HPG layer for tailored selectivity towards specific applications. Furthermore， a systematic evaluation of its performance using complex biological and environmental matrices will be conducted to assess its practical applicability.

褐藻胶是一种主要从褐藻中提取的酸性多糖^［1］^，具有良好的凝胶特性，在食品领域中通常用作增稠剂和稳定剂^［2］^，此外褐藻胶还是一种膳食纤维，可以改善代谢紊乱等问题^［3］^。然而，褐藻胶分子质量高、凝胶性强、不易被机体吸收等特性限制了其在活性方面的应用^［4］^。褐藻胶寡糖（alginate oligosaccharide， AOS）是褐藻胶的降解产物。相比于褐藻胶，AOS具有优异的抑菌性^［5］^、抗肿瘤性^［6］^、抗炎^［7］^和增强机体免疫功能^［8］^等生物活性功能。良好水溶性更是促进了AOS在保健品、化妆品、医药、有机肥料、功能食品等行业领域中的应用^［9］^。

众多研究表明，AOS是由古罗糖醛酸（guluronic acid， G）和甘露糖醛酸（mannuronic acid， M）单体聚合而成。仅由G单体构成的古罗糖醛酸寡糖可以调节褐藻胶溶液的流变特性，作为黏合剂形成褐藻胶溶液凝胶^［10］^。由M单体组成的甘露糖醛酸寡糖具有保护神经系统的功能，而且甘露糖醛酸寡糖衍生物还表现出了降血脂以及降血糖等功效^［11-13］^。在聚合度为2~5的AOS中，只有聚合度为5的AOS对骨肉瘤细胞表现出抗肿瘤作用，该聚合度的AOS可有效提升血清中高密度脂蛋白胆固醇以及谷胱甘肽的含量，增强人体的抗氧化能力^［1，14］^。目前，传统的色谱分离方法如离子交换色谱、凝胶渗透色谱等无法得到单一聚合度和高纯度的AOS，限制了其在分子水平上的应用^［15，16］^。为了获得组分单一、结构明晰的AOS，并阐明其结构-功能关系，有效分离AOS已成为必要前提^［17］^。亲水作用色谱（HILIC）近年来在糖类化合物的分离分析中得到了广泛关注。HILIC以极性固定相和高比例有机相-水相混合流动相为特点，主要基于溶质在固定相表面水层和流动相之间的相互作用进行分离^［18］^。与传统的反相色谱相比，亲水作用色谱更适合分离极性化合物，能够提供与反相色谱不同的分离选择性。对于极性较强的褐藻胶寡糖，亲水作用色谱有望实现更好的分离效果。

超支化聚甘油（hyperbranched polyglycerol， HPG）是由甘油通过聚合反应得到的高分子聚合物。HPG以甘油为基本结构单元，通过羟基之间的缩聚反应形成高度支化的三维立体结构^［19］^。在聚合过程中，甘油分子中的羟基不断发生反应，形成大量的支链和末端羟基，使得分子具有较高的分子质量和独特的树枝状结构^［20］^。因此，HPG在水中和多种有机溶剂中具有良好的溶解性，分布在分子表面和内部的羟基赋予其高反应活性和功能化潜力。

本研究利用HPG的高亲水性和生物相容性，设计并制备了一种新型HPG功能化极性固定相（HPG-Sil）。该固定相通过二氧化硅表面的硅醇基与HPG间的聚合反应，形成具有高密度羟基和分子内氢键的HPG主链，提升固定相亲水性和分离性能，并将HPG-Sil应用于AOS的分离。本研究为高纯度、单一聚合度AOS的制备及其结构-功能关系研究提供新的方法和技术支撑，同时拓展了HPG在高效液相色谱模式下的应用潜力。

## 1 实验部分

### 1.1 仪器、试剂与材料

3200L色谱仪（大连依利特分析仪器有限公司）；STA449F3热重分析仪（TGA，德国Netzsch公司）；SU8000场发射扫描电子显微镜（SEM，日本Hitachi Ltd公司）；Tecnai G2 Spirit透射电子显微镜（TEM）、MULTIFUGE X1R高速冷冻离心机（美国Thermo Fisher Scientific公司）；Spectrum 3傅里叶变换红外光谱仪（FTIR，美国PerkinElmer Instruments公司）；Vario EL cube元素分析仪（德国Elementar Analysensysteme公司）；ASAP-2020氮气吸附仪（美国Micromeritics公司）；DZF-6020真空干燥箱（上海精宏实验设备有限公司）。

硅胶（5 μm）购于苏州纳微股份有限公司；甲醇钠（纯度97%）、缩水甘油（纯度97%）、分子筛（4A）均购于上海阿拉丁生化科技股份有限公司；胸腺嘧啶、尿嘧啶、次黄嘌呤、腺苷（色谱纯，购于北京J&K科学有限公司）；氨水（色谱纯）、甲苯（分析纯）、甲醇（色谱纯）、甲酸铵（色谱纯）、丙酮（色谱纯）、醋酸（色谱纯）、偶氮异丁腈（AIBN，纯度98%）均购于上海麦克林生化科技股份有限公司；乙腈（色谱纯）与无水乙醇（色谱纯）购于上海泰坦科技有限公司。褐藻胶寡糖由中国海洋大学制备和提供。

### 1.2 多羟基硅胶固定相HPG-Sil的制备

HPG-Sil微球的制备过程见图1。取5.0 g硅胶于浓盐酸中浸泡活化24 h，过滤后用水洗涤至中性，于80 ℃鼓风干燥箱中干燥过夜。

**图1:**
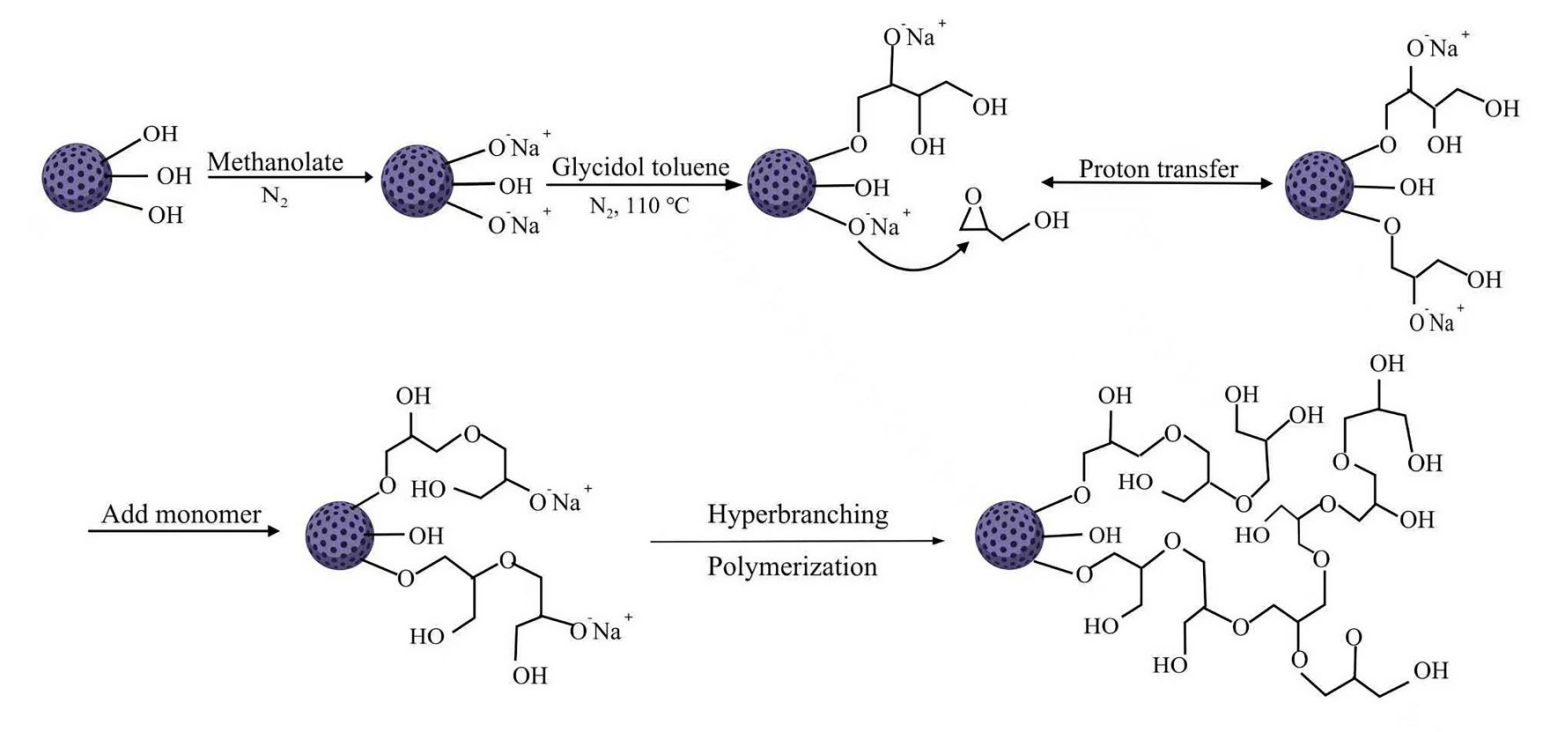
HPG-Sil的合成路线

将4.0 g活化完全的硅胶放入250 mL烧瓶中，在氮气气氛下加入45 mL甲醇和2.16 mL甲醇钠溶液，超声5 min获得均匀悬浮液，并在室温下以250 r/min搅拌30 min，过滤，并用100 mL无水甲醇和甲苯洗涤。硅胶在100 ℃下真空干燥30 min。在氮气气氛下，加入48 mL干燥后甲苯和16 mL缩水甘油，超声处理得到均匀的悬浮液。悬浮液在110 ℃下以300 r/min搅拌40 min，通过G4烧结玻璃漏斗过滤，依次用乙醇、水和甲醇洗涤。将过滤固体在60 ℃下干燥过夜，得到修饰1层HPG的固定相HPG-Sil 1。重复以上步骤，得到修饰3层HPG的固定相HPG-Sil 3。

### 1.3 色谱柱的填装

空柱管的前处理：用无水乙醇将空柱管清洗干净，再以热风吹干柱管。

用于分离的色谱柱装填过程如下：取1.38 g上述制备的HPG-Sil 3固定相超声分散于甲醇中，以甲醇为顶替液，在50 MPa下将固定相填充到不锈钢管（150 mm×4.6 mm， 大连依利特分析仪器有限公司），关闭阀门将压力缓慢降至40 MPa维持30 min后自然泄压，压力表显示为0后，拆下色谱柱，观察填料是否填装整齐，盖上筛板，旋紧柱帽。

### 1.4 样品制备

胸腺嘧啶、尿嘧啶、次黄嘌呤和腺苷4种标准品分别称取0.3 mg超声溶解于10.0 mL乙腈-水（85∶15，体积比）中，经0.22 μm微孔滤膜过滤后得到标准样品溶液。

称取2.5 mg AOS样品超声溶解于10.0 mL乙腈-水（85∶15，体积比）中，经0.22 μm微孔滤膜过滤后得到AOS样品溶液。

## 2 结果与讨论

### 2.1 HPG-Sil固定相的表征

通过红外表征方法对HPG-Sil 3固定相表面官能团结构进行分析。在HPG-Sil微球的红外图谱中（图2），2 953 cm^-1^和2 890 cm^-1^处的峰对应C-H键的反对称伸缩振动和对称伸缩振动。而在原始硅球的红外图谱中则没有这些特征峰的出现。支化聚甘油分子中含有大量醚键（C-O-C）和羟基（C-OH）。图2中显示HPG-Sil 3在1 000~1 200 cm^-^¹处的红外吸收增强，说明HPG分子中C-O键增多。以上红外光谱结果表明超支化聚甘油在硅球表面的成功接枝。

**图2:**
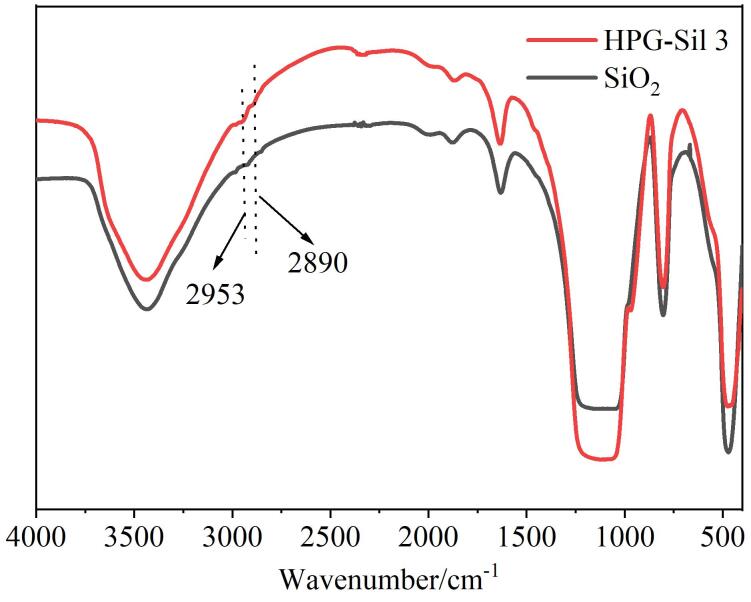
SiO_2_和HPG-Sil 3的红外光谱图

载碳量常用于表征硅胶表面化学修饰的程度，碳含量会影响固定相对分析物的吸附强度^［21］^。采用元素分析仪对接枝不同层数HPG硅球中的C、H元素含量进行测定，结果表明（表1），HPG-Sil 3的碳含量是SiO_2_的20倍，HPG-Sil 3的氢含量是SiO_2_的1.8倍，因此说明超支化聚甘油已成功键合在硅胶表面。HPG-Sil 3的表面键合度是HPG-Sil 1的2.3倍，说明HPG-Sil 3固定相具有更高的HPG接枝密度。

**表1:** SiO_2_和接枝不同层数HPG-Sil固定相的元素分析

Analyte	Elemental contents/%		Surface coverage/（μmol/m^2^）
	C	H	
SiO_2_	0.25	0.367	/
HPG-Sil 1	2.22	0.598	1.41
HPG-Sil 3	4.9	0.673	3.30

为了深入探究HPG在HPG-Sil 3固定相上的键合情况，采用TGA对其进行热重分析，见图3。原始硅球和HPG-Sil 3固定相在温度低于200 ℃时的失重现象主要源于物理吸附水的挥发；200~800 ℃时，原始硅胶和HPG-Sil 3固定相的质量损失分别为2.88%和7.46%。热重损失率的提高说明HPG在HPG-Sil 3固定相上成功键合。

**图3:**
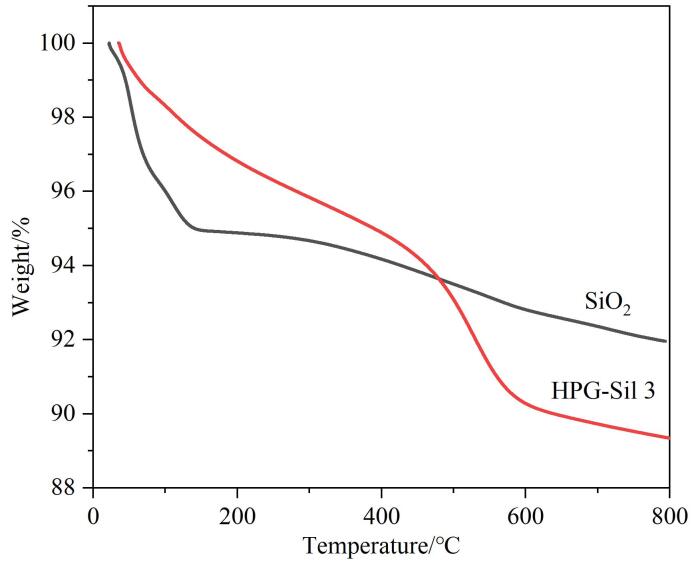
SiO_2_和HPG-Sil 3的热失重曲线

SiO_2_和HPG-Sil 3固定相的SEM和TEM结果如图4所示。通过对比图4a~4c可以看出，经过超支化聚合物接枝后的HPG-Sil 3微球的表面形态与SiO_2_相似，都是均匀球形结构，微球之间没有聚集现象，呈现单分散状态，说明超支化聚甘油改性后微球的形态良好。此外，从图4c中可以看出，HPG-Sil 3固定相表面光滑，说明聚合物均匀键合在二氧化硅微球的表面以及孔内。从图4e可观察到颗粒表面显示出与内部不同的衬度，说明颗粒表面存在特殊的化学修饰，即硅球表面存在修饰层。由图4f可以看出，HPG-Sil 3固定相的多孔结构，说明合成得到的HPG-Sil 3固定相仍保持良好的多孔结构。

**图4:**
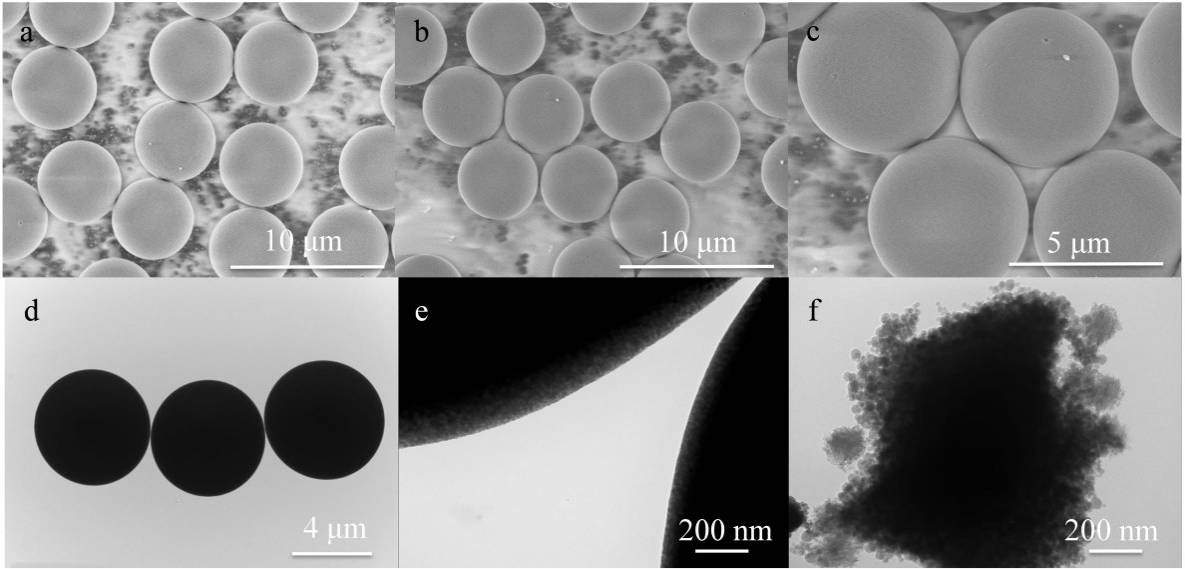
（a）SiO_2_、（b，c）HPG-Sil 3的SEM图以及（d）SiO_2_、（e，f）HPG-Sil 3的TEM图

图5显示了SiO_2_和HPG-Sil 3固定相的氮气吸附-脱附等温线和孔径分布图。从图中可以看出，HPG-Sil 3的氮气吸附-脱附等温线和SiO_2_固定相同属于典型的Ⅳ型，说明改性后硅球的介孔结构没有受到破坏，且微球内部孔道相互连通，具有很好的通透性；从孔径分布图可以发现，接枝官能团后的硅球孔径分布均匀，其大小与SiO_2_相比稍有减小，说明聚合物均匀键合到二氧化硅微球的表面和孔道内部。表2列出了SiO_2_和HPG-Sil 3微球的比表面积、孔体积和孔径数据。硅球表面引入HPG后，孔体积和孔径分别从1.07 cm³/g、12.57 nm下降到0.95 cm³/g和9.33 nm，比表面积也从458.41 m²/g下降到301.13 m²/g，这可能是因为硅胶接枝了HPG官能团，部分孔道被HPG官能团占据。因此，HPG-Sil 3固定相的孔体积和孔径略有减小，但保持了良好的介孔结构。

**图5:**
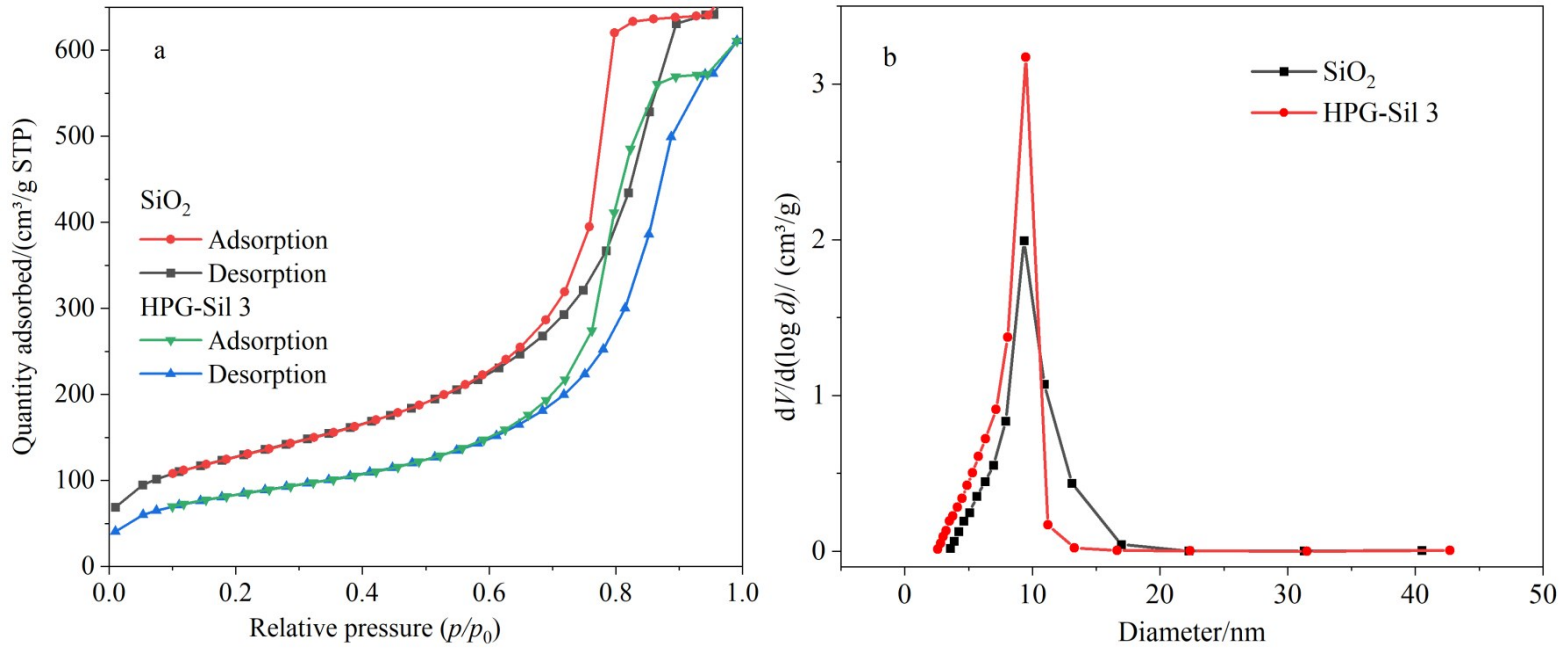
（a）SiO_2_和HPG-Sil 3的氮气吸附-脱附等温线图以及（b）SiO_2_和HPG-Sil 3的孔径分布图

**表2:** SiO_2_和HPG-Sil 3固定相的比表面积、孔体积和孔径

Analyte	Specific surface area/（m²/g）	Pore volume/（cm³/g）	Pore diameter/nm
SiO_2_	458.41	1.07	12.57
HPG-Sil 3	301.13	0.95	9.33

### 2.2 HPG-Sil与SiO_2_固定相的分离效果对比

胸腺嘧啶（辛醇比lg *P*=-0.4）、尿嘧啶（lg *P*=-0.45）、次黄嘌呤（lg *P*=-0.11）和腺苷（lg *P*=-1.05）都属于结构明确的小分子标准品，利用这4种典型的亲水性化合物作为分析物品能够快速、全面地评估固定相的性能。

如图6所示，4种分析物不能被SiO_2_柱很好地分离，出现了两对分析物的共洗脱。相比SiO_2_柱，分析物的分离效果在HPG-Sil 1柱上轻微改善，样品的保留时间、分离度和理论塔板数均增加，峰形的拖尾情况也得到了较好改善。分析物在HPG-Sil 3柱中呈现出更好的分离和更高的柱效（48 000~64 000 N/m），相邻两色谱峰的分离度为1.4~9.44，色谱峰的拖尾因子介于0.79~0.85，色谱峰对称性较好。由于胸腺嘧啶和尿嘧啶的保留较弱，这两种物质最先被分离出来。核苷中糖环的存在增加了分子的极性，两个核苷（次黄嘌呤和腺苷）在碱基（胸腺嘧啶和尿嘧啶）之后洗脱。分析结果表明，HPG-Sil 3固定相中富含羟基基团，实现了4种极性溶质在短时间内高效且稳定的分离，而且由出峰顺序和洗脱机理可得，该色谱固定相符合亲水相互作用机理。由于模型分析物的保留时间、理论塔板数和分离度随HPG层数的增加而增加，拖尾因子逐渐靠近1.0，因此对HPG-Sil 3柱的分离模式进行进一步的研究。

**图6:**
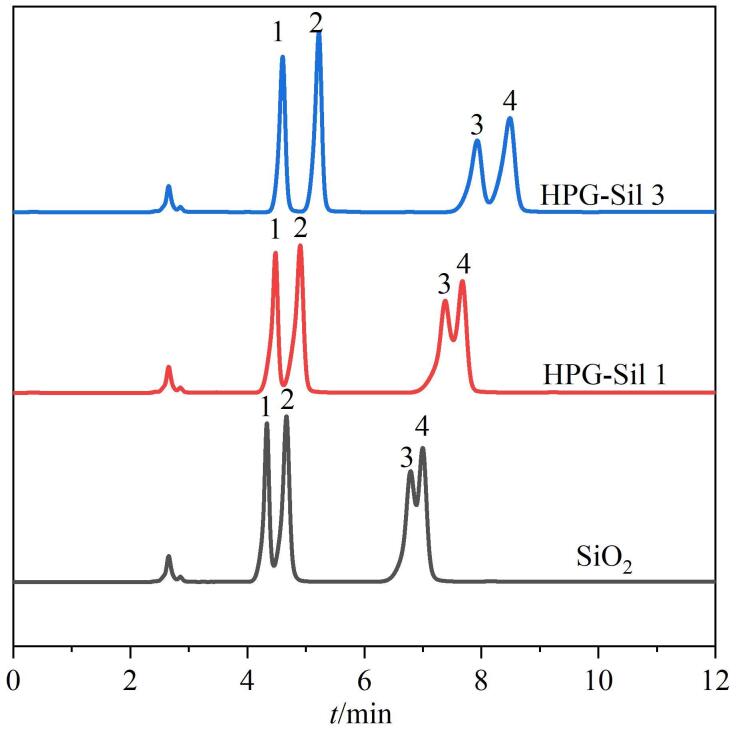
采用不同固定相色谱柱时分析物的色谱图

### 2.3 HPG-Sil 3固定相色谱行为研究

#### 2.3.1 流动相中乙腈含量对分析物保留的影响

洗脱液的溶剂强度对分析物的保留有重要影响。以胸腺嘧啶、尿嘧啶、次黄嘌呤和腺苷为分析物，研究了流动相中乙腈含量对极性分析物在HPG-Sil 3固定相上的保留行为。如图7所示，随着流动相中乙腈含量增加，分析物在固定相上的保留时间延长，*k*呈上升趋势，当乙腈体积分数为85%时，分析物的分离度和理论塔板数达最大值。这是因为随着流动相有机溶剂所占体积分数增加，极性分析物与HPG-Sil 3固定相表面的极性基团以及水层之间的相互作用增强，保留增强，符合亲水作用色谱分离模式。

**图7:**
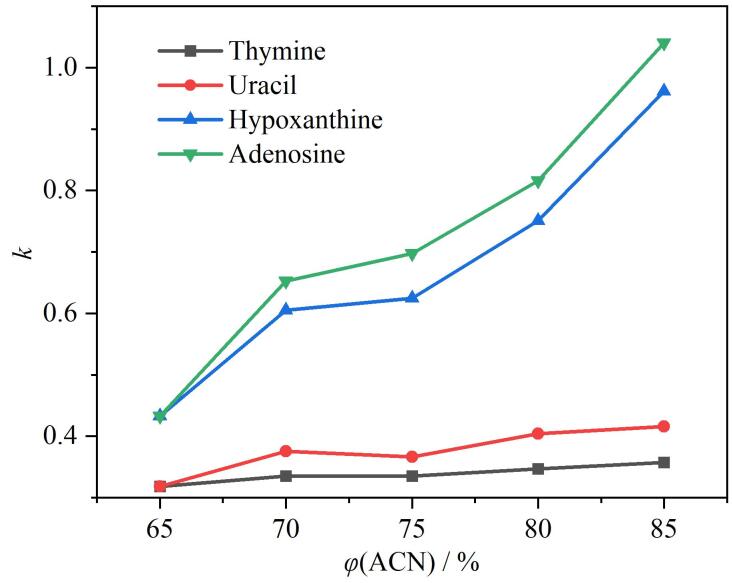
乙腈体积分数对分析物在HPG-Sil 3固定相上保留因子的影响

#### 2.3.2 流动相中缓冲盐浓度对HPG-Sil 3固定相保留行为的影响

在HILIC模式下，流动相中缓冲盐的浓度对极性化合物尤其是离子型化合物的保留行为会产生重要影响。缓冲盐通过调控分析物在流动相和固定相表面富水层之间的分配以及分析物与固定相之间的电荷作用等方式调节极性分析物的保留性质和选择性^［22］^。本文研究不同浓度的甲酸铵缓冲液对HPG-Sil 3分离极性标准品的影响。由图8可见，对于胸腺嘧啶、尿嘧啶、次黄嘌呤和腺苷，保留因子均随着流动相中甲酸铵浓度的增加而增加，这是因为随着流动相中缓冲盐浓度的增加，缓冲盐会向固定相表面富水层迁移，增加富水层的亲水性，从而增强极性溶质的保留并提高分离度，溶质的流出顺序与其辛醇比数值变化一致，说明分离过程服从分配机理。同时，随着缓冲盐浓度的提高，增加了流动相中离子强度，减小了静电作用引起的峰前延并使峰宽变窄，从而起到改善峰形的作用，使柱效增加。因此，流动相中提高缓冲盐浓度可增强HPG-Sil 3固定相对极性分析物的保留。

**图8:**
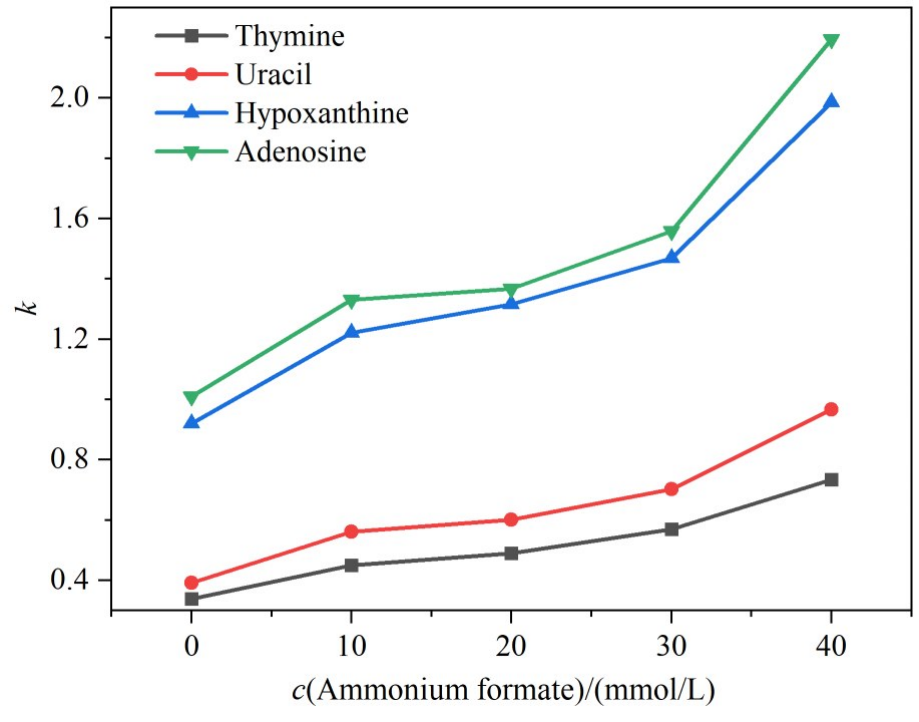
流动相中甲酸铵浓度对分析物在HPG-Sil 3固定相上*k*的影响

#### 2.3.3 流动相pH值对HPG-Sil 3固定相保留行为的影响

在HILIC模式下，流动相pH值会改变固定相和溶质的电荷状态，从而影响带电固定相与带电溶质之间的静电相互作用（吸引或排斥），进而影响分析物的保留性^［23］^。当固定相与极性分析物所带电荷相同时，由于静电排斥作用，分析物的保留会发生减弱；反之则保留增强。在此研究中，流动相中乙腈-水（85∶15，体积比），甲酸铵浓度恒定为40 mmol/L，研究了流动相pH在3.9、4.7、5.5、6.5、7.5时对极性分析物保留行为的影响。如图9所示，在HPG-Sil柱上，发现随着流动相pH的增加，胸腺嘧啶（p*K*_a_=9.9）、尿嘧啶（p*K*_a_=9.5）、腺苷（p*K*_a_=13.2）和次黄嘌呤（p*K*_a_=8.7）的保留因子均呈现不断上升的趋势，当pH值较高的情况下，具有更高的理论塔板数和分离度。随着pH值增加，固定相表面的羟基电离程度增强，但不会影响分析物的电离状态，导致固定相与分析物之间的离子交换作用增强，因此分析物的保留因子呈现不断上升的变化规律。因此，在HILIC模式下，极性分析物在固定相HPG-Sil 3上保留受其在流动相和固定相表面分配作用及离子交换作用共同影响。

**图9:**
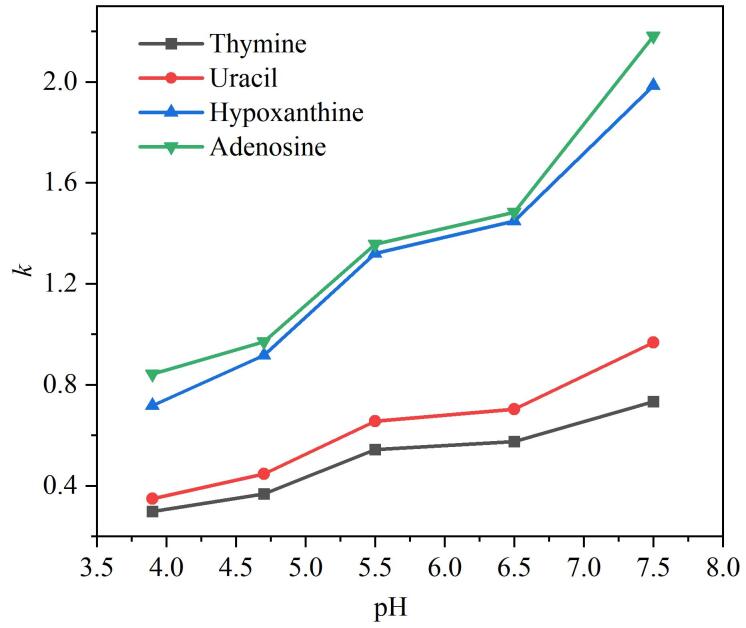
流动相缓冲液pH对分析物在HPG-Sil 3固定相上*k*的影响

#### 2.3.4 HPG-Sil 3固定相分离极性化合物的机理

自HILIC保留机理模型是基于分析物在固定相上的“富水层”和流动相间的分配。分析物由于在水层和流动相中分配系数不同而实现分离^［24］^，表示分配作用机理的理论模型如式（1）所示^［25］^：


lg k=A-Sφ （1）

式中：*A*为常数；*k*为溶质的保留因子；*S*为所得lg *k*对*φ*线性拟合时曲线的斜率；*φ*为流动相中强洗脱溶剂（水）的体积分数（%）。

虽然分配机制被大多数人所接受，但是很多实验结果不能被该机制所解释。后来提出亲水色谱的机理符合正相色谱的吸附机理^［26］^。该机理被用来表征吸附作用的理论模型，如式（2）所示^［27］^：


lg k=B-nlg φ （2）

式中： n 为lg *k*对lg *φ*线性拟合时曲线的斜率。

在HILIC色谱中的多重保留机理是由于固定相表面形成了“富水层”，分析物在固定相之间存在分配作用，同时由于固定相表面官能团的不一致性，分析物与固定相表面之间往往还存在氢键等作用。该理论模型中分析物的保留因子与流动相中水的含量之间的关系如式（3）所示^［28］^：


ln k=a+bφ-cln φ （3）

式中：*a*一般为常数；*b*是与溶质和固定相直接作用相关的系数；*c*是溶质与溶剂之间的相互作用相关的系数。

流动相中水含量作为影响分析物保留的重要因素，其含量变化直接影响流动相极性强弱，从而改变分析物与“富水层”的分配作用和与固定相间的吸附作用大小。通过样品的保留因子和流动相含水量间的关系，研究HPG-Sil 3固定相分离极性标准品的机理。使用分配、吸附和多重相互作用模型对4种极性标准品的保留因子与流动相中水含量进行拟合，拟合曲线如图10所示。

**图10:**
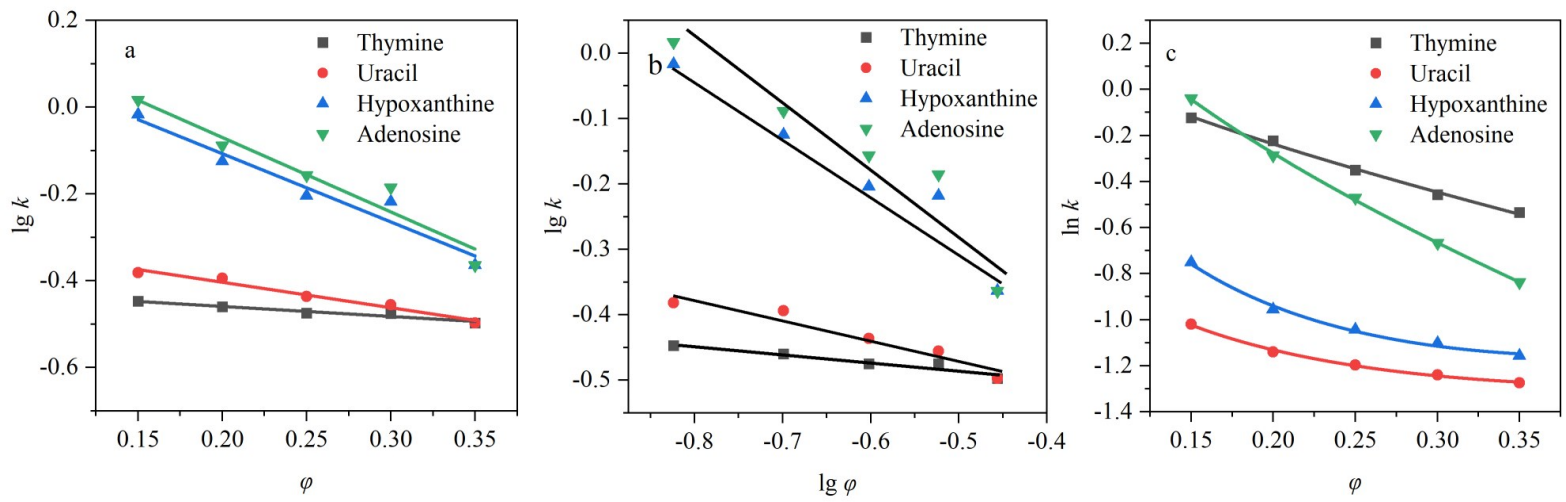
HPG-Sil 3固定相在亲水模式下的保留及分配评价

４种极性标准品的分配机理回归系数*r* ^2^为0.979~0.998，吸附机理回归系数*r* ^2^为0.954~0.989，多重保留机理拟合度*r* ^2^均大于0.996，说明极性样品在HPG-Sil 3固定相对极性物质的保留符合多重保留机理，既有色谱分配机理，又有以氢键相互作用为主导的色谱吸附机理，且分配作用大于吸附作用。

### 2.4 HPG-Sil 3在褐藻胶寡糖分离中的应用

在优化的分离条件下，HPG-Sil 3柱分离褐藻胶寡糖的色谱图如图11所示。结合文献［^29^，^30^］中的色谱图可知，HPG-Sil 3固定相在亲水模式下成功分离出聚合度为2~7的褐藻胶寡糖。HPG-Sil 3固定相的分离作用主要基于其表面的超支化聚合物结构。HPG具有丰富的羟基和三维网络结构，能够与褐藻胶寡糖分子中羧基上的羰基氧原子和羟基氢原子结合形成氢键。当褐藻胶寡糖和HPG固定相两个羟基距离较近时，一个羟基中的氧原子电负性较大，会吸引另一个羟基中氢原子上的电子，使氢原子带有部分正电荷，氧原子带有部分负电荷，从而产生静电相互作用。这些相互作用使得不同聚合度的褐藻胶寡糖在固定相上的保留时间不同，从而实现了有效分离。从图11中可以清晰地观察到不同聚合度的寡糖峰形尖锐且分离度良好，色谱图中各寡糖峰的对称性和尖锐性进一步说明了该固定相在分离过程中具有较低的传质阻力和良好的柱效。因此HPG-Sil 3对褐藻胶寡糖具有较高的选择性和分离能力。

**图11:**
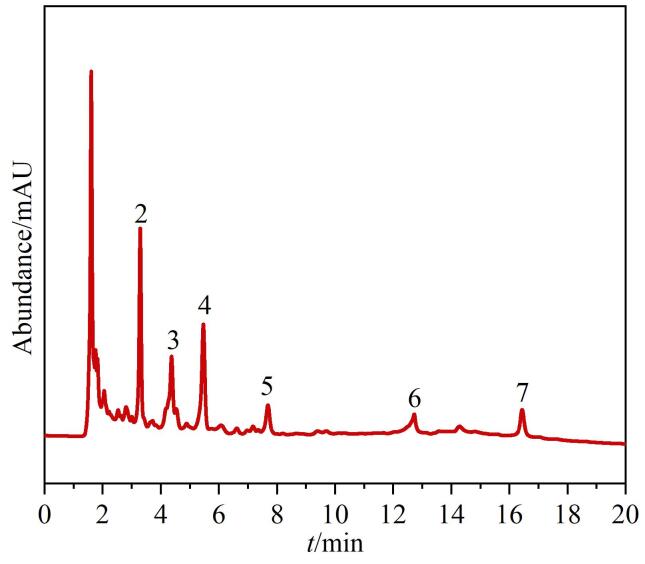
优化条件后HPG-Sil 3固定相分离褐藻胶寡糖的色谱图

色谱固定相的重复性和稳定性是评估其质量的重要参数。以褐藻胶寡糖为测评分子，在每日进样6针，每日每针中间间隔15 min，连续进行实验5天，考察HPG-Sil 3固定相的日内和日间重复性。聚合度为2~7的褐藻胶寡糖日内和日间保留时间的相对标准偏差为0.20%~0.78%（*n*=6）和0.35%~0.43%（*n*=5），说明该色谱固定相具有良好的稳定性和分离重复性。

## 3 结论

本研究制备了超支化聚甘油接枝固定相HPG-Sil 3，研究了其对极性化合物的保留机理，并成功应用于褐藻胶寡糖的分离。后续工作可进一步拓展该固定相在不同寡糖分离中的应用，探索其在复杂生物样品中寡糖分离的潜力；优化固定相制备工艺，提高制备效率和质量稳定性；结合新型检测技术，提升对寡糖分离检测的灵敏度和准确性；深入研究固定相的作用机制，为进一步优化色谱条件和固定相性能提供理论依据。
